# Alignment of physical education curricula with physical literacy across Europe: an observational mapping study with country-level predictors

**DOI:** 10.1016/j.lanepe.2026.101641

**Published:** 2026-03-10

**Authors:** Johannes Carl, Kasper Salin, Lisa M. Barnett, Lawrence Foweather, Gregor Jurak, João Martins, Ivan Müller, Wesley O'Brien, Fotini Venetsanou, Amika Singh, Peter Elsborg, Hannah Goss, Suzanne Lundvall, Iuliia Pavlova, Cristiana D'Anna, Petr Vlček, EUROPLIT group, Beatrix Algurén, Branislav Antala, Gillian Bartle, Jens Birch, Anna Bryant, Jorge Carlos-Vivas, Efstathios Christodoulides, Joe G. Cowley, Tamás Csányi, Kristine De Martelaer, Arunas Emeljanovas, Gonca Eren, Andra Fernāte, Barbara Gilic, Thordis Gisladottir, Nigel Green, Dorota Groffik, Sandra Heck, Ivo van Hilvoorde, Mikko Huhtiniemi, Teodora-Mihaela Iconomescu, Johannes Jaunig, Ellen Jones, Anne Kelso, Andre Koka, Christoph Kreinbucher-Bekerle, Ida Laudańska-Krzemińska, Bojan Masanovic, Paul McFlynn, Melanie McKee, María Mendoza-Muñoz, Paulina S. Melby, Brigita Mieziene, Ivana Milanović, Eleonora Mileva, Alexandre Mouton, Zsolt Németh, Bogdan Sorin Olaru, Marcos Onofre, Petro Petrytsa, Maret Pihu, Biljana Popeska, Stevo Popovic, André Poweleit, Snežana Radisavljević Janić, Vassiliki Riga, Christophe Schnitzler, Damir Sekulic, Marina Semyonova, Baiba Smila, Clemens Töpfer, Olia Tsivitanidou, Jana Vašíčková, Øystein Winje, Viviana Zito, Günay Yıldızer

**Affiliations:** a Deakin University, Institute for Physical Activity and Nutrition, School of Health and Social Development, Geelong, VIC, Australia; b University of Jyväskylä, Faculty of Sport and Health Sciences, Jyväskylä, Finland; c Liverpool John Moores University, Research Institute for Sport and Exercise Sciences, Liverpool (Merseyside), England, United Kingdom; d University of Ljubljana, Faculty of Sport, Ljubljana, Slovenia; e Universidade de Lisboa, Instituto de Educação, UIDEF e Faculdade de Motricidade Humana, Lisboa, Portugal; f Department of Sport, Exercise and Health, University of Basel, Basel, Switzerland; g University College Cork, Physical Education, Sports Studies and Arts Programme, Cork, Ireland; h National and Kapodistrian University of Athens, School of Physical Education and Sport Science, Dafni/Athens, Greece; i Mulier Institute, Utrecht, Netherlands; j Windesheim University of Applied Sciences, Zwolle, Netherlands; k Frederiksberg Hospital, Center for Clinical Research and Prevention, Frederiksberg, Denmark; l Dublin City University, School of Health and Human Performance, Dublin, Ireland; m Department of Food and Nutrition and Sport Science, University of Gothenburg, Gothenburg, Sweden; n Department of Sport, Food and Natural Sciences, Western Norway University of Applied Sciences (HVL), Sogndal, Norway; o Department of Theory and Methods of Physical Culture, Ivan Boberskyj Lviv State University of Physical Culture, Lviv, Ukraine; p Department of Psychology and Education, Pegaso University, Naples, Italy; q Goethe University Frankfurt, Institute of Sports Sciences, Frankfurt am Main, Germany

**Keywords:** Children, Curriculum, Exercise, Physical activity, Schools, Teaching, Wellbeing

## Abstract

**Background:**

Physical literacy (PL) is recognised by UNESCO and WHO for fostering lifelong physical activity. Although PL stands on the global agenda of health and education policies, analyses of the adoption by European education are scant. This study aimed to map the alignment of physical education with PL in Europe, explore potential predictors at country level, and identify country ‘alignment laggards’.

**Methods:**

Experts from 40 European countries assessed the alignment of national physical education curricula with PL using a pre-validated 15-indicator survey (α = 0·86). Country-level PL alignment scores were calculated and mapped. Regression models investigated whether these scores were predicted by educational attainment (PISA), human development (Human Development Index), relative economic strength (Gross Domestic Product/Capita), societal liberty (Human Freedom Index), and innovation spirit (Global Innovation Index). Residual errors were computed to identify association-adjusted ‘laggards’ in curricular PL alignment.

**Findings:**

Significant heterogeneity in PL alignment existed across Europe. Estonia, Wales, Finland, and Norway showed the highest, and Romania, Croatia, Cyprus, and England the lowest PL alignment. Countries' educational attainment (*β* = 0·43 [CI_95_ 0·13, 0·65], *p* = 0·0062), human development (*β* = 0·32 [CI_95_ 0·011, 0·58], *p* = 0·043), and innovation spirit (*β* = 0·32 [CI_95_ 0·0071, 0·58], *p* = 0·046) were significantly associated with curricular PL alignment but not relative economic strength and liberty. A total of 17 European countries were consistent ‘laggards’ in curricular PL alignment across all significant predictors.

**Interpretation:**

PL policies follow educational, developmental, and innovative gradients. Stakeholders should integrate PL as a strategic lever to transform physical education, guiding curricula and programmes to foster health through lifelong physical activity.

**Funding:**

None.

**Research in context**

**Evidence before this study**

Global health and education policies from WHO and UNESCO highlight the need for physical education to address physical literacy (PL) to promote lifelong physical activity. We searched PubMed, Scopus, EBSCO (7 sub-databases), and ProQuest (14 sub-databases) for literature reviews published up to 16 June 2023 (no restriction in the start date) to identify “blank sports” in the PL literature [34], using search terms related to “physical literacy”, and “review”. No language or start-date restrictions were applied. We screened 4830 articles (including reference lists), and 41 reviews were integrated based on four eligibility criteria (PL relevance, academic journal, language, and study type) following independent rating. Supported by PRISMA guidelines and study quality assessment via the AMSTAR-2 tool, this published review [34] revealed a notable absence of policy studies or cross-national analyses quantifying whether/how effectively national physical education curricula across Europe operationalise the PL concept. Confirmed through continuous monitoring of database alerts and targeted searches up to 19 February 2026, this research gap persisted, with no new cross-national policy analyses addressing these specific PL operationalisation metrics in Europe.

**Added value of this study**

This is the first participatory study to use a validated instrument to systematically map and quantify the curricular alignment with PL policy across 40 European countries. The findings identify significant regional heterogeneity and reveal that curricular PL alignment strongly follows countries’ educational attainment, human development, and innovative gradients (but not economic strength and societal liberty gradients in our analyses).

**Implications of all the available evidence**

This country-level mapping suggests that the integration of global PL policy is dependent on national developments, regional contexts, and educational frameworks. The evidence identifies specific European countries as ‘alignment laggards’ where a policy focus on PL could offer a significant public health gain by bridging the gap between existing curriculum content and global recommendations.

## Introduction

Physical education is a cornerstone subject in schools that aims to empower individuals for lifelong physical activity.^1^^,^^2^ Participation in physical education is recognised as a human right and reaches many children worldwide, with 83% of all countries reporting physical education as a compulsory school subject.^1^^,^^3^ When aligned appropriately, physical education has the potential to make a substantial contribution to individual and public health.^4^^,^^5^ In 2015, the United Nations Educational, Scientific and Cultural Organization (UNESCO) released its physical education standards to increase both the quantity and quality of physical education provision across the globe.^1^ These standards gave the concept of physical literacy (PL) a prominent role, concluding that the “promotion of physical literacy should […] remain a key feature of any physical education curriculum throughout primary and secondary education”.^1^ Similarly, the World Health Organization (WHO) stated in its Global Action Plan on Physical Activity (GAPPA) 2018–2030 that the “provision of quality physical education and supportive school environments can impart physical and health literacy for lifelong healthy, active lifestyles”.^6^ As highlighted in the statements by UNESCO and WHO, PL increasingly stands on the global agenda to harness the potential for lifelong physical activity through physical education.^7^

Aiming to move beyond narrow focuses on sport, fitness, and performance, PL targets holistic development in physical education^8^^,^^9^ by strengthening the “physical, psychological, cognitive, and social capacities to support health-promoting and fulfilling movement and physical activity—relative to their situation and context—throughout their lifespan”.^10^ Although there are slightly different definitions and understandings of PL globally (e.g., in Australia, Canada, China, England, or by the International Physical Literacy Association),^11^ all definitions seek to transform children's physical activity towards more sustained engagement and, therefore, the implementation of PL in the school context has great potential.^12^^,^^13^ The integration of physical, social, cognitive, and affective experiences through physical activity intends to bridge the common separation between body and mind^14^ and to more effectively meet the diverse needs and preferences of all students, thereby enhancing the public health potential of physical education.^15^ Indeed, a recent study demonstrated that the effect of quality physical education on students' physical activity behaviour is mediated through PL.^16^ According to a model by Cairney et al., there is a direct pathway from PL via enriched physical activity to biopsychosocial health.^8^ Two meta-analyses revealed that PL is linked to higher physical activity levels and that related interventions are more effective than conventional exercise interventions in improving physical activity levels, as well as physical, affective, and cognitive outcomes.^17^^,^^18^ Given these empirical findings, PL could make a significant contribution to combat the rise in physical inactivity among adolescents— a key problem for global health across societies.^19^ Notably, researchers have highlighted the potential of PL not only to enhance physical activity and health, but also to positively influence broader developmental outcomes. First, researchers have argued that PL-informed practices place greater emphasis on students' cognitive engagement and utilisation of creative solutions when compared to practices focused solely on physical performance.^20^^,^^21^ As such, PL-informed practices may help amplify the known cognitive and academic benefits of physical activity.^22^^,^^23^ Second, researchers suggest that PL can even contribute to human flourishing, positive youth development, biopsychosocial health, and better quality of life.^8^^,^^24^, ^25^, ^26^ Supporting this, two cross-sectional studies have found positive associations between individuals' PL and quality of life indicators.^27^^,^^28^

Despite the compelling individual-level evidence regarding the beneficial effects of PL and the widespread recognition of the importance of addressing physical, affective, cognitive, and social dimensions in physical education,^29^^,^^30^ the scientific literature lacks a comprehensive overview of the global adoption of PL into physical education policies and curricula. While recent research has focused on conceptual, psychometric, and interventional aspects of the concept,^13^^,^^31^, ^32^, ^33^ a review of reviews has highlighted the absence of policy studies and cross-national analyses on PL.^34^ Consequently, the current body of knowledge on the adoption of PL in Europe does not match with its macropolitical relevance of PL, as emphasised by UNESCO and the WHO.

This study had three primary objectives. First, it aimed to quantify and map the extent to which physical education curricula in European countries align with UNESCO's and WHO's expectations for PL using country-level data. In line with a comprehensive understanding of curriculum analyses,^35^ the study defines the alignment with UNESCO and WHO by the degree of how agreed principles of PL^36^ permeate the goals, content, management, and methods of formal physical education. Second, it examined national characteristics that may support stronger alignment with PL principles in physical education. From an education and health policy perspective, it would be critical to know whether the curricular adoption of PL may depend on the general educational capacity of a nation (e.g., curriculum depth and teacher qualifications), on its human development (e.g., focus on general literacy, stable governance structures, and a commitment to human flourishing and biopsychosocial health), on its economic strength (e.g., financial capacity for regular curriculum reform and resource allocation), on its societal liberty (e.g., focus on individual empowerment, human rights, holistic development, and autonomy values), and on its innovative spirit (e.g., focus on creative outputs, international developments, and knowledge absorption capacity).^8^^,^^25^^,^^37^^,^^38^ Using an exploratory approach, this study investigated whether curricular PL alignment is associated with a country's educational standards, level of human development, relative economic strength, degree of liberty, and innovative spirit. Third, it sought to identify European countries with high potential for improved PL alignment (so-called “alignment laggards”) in physical education.

## Methods

### Study design

This study marked a collaborative research effort along five stages. In the first stage, the core team (JC, KS) identified PL experts for the European countries (one per country) and invited them via e-mail to participate in collaboratively surveying PL. The experts were recruited through a network on PL that has promoted cross-country exchange on this topic since 2021 and that has grown in recent years (for details, see Carl and colleagues^36^ and especially Supplementary Material 1). For inclusion in this study, we defined an ‘expert’ as a person who simultaneously fulfilled the following^39^: (a) member of an academic institution (e.g., university or scholarly society), (b) specialist in physical education or physical activity promotion, and (c) knowledge and understanding of PL. In addition, at least one person per country had to be an expert in physical education for school children to ensure pedagogical expertise (additional requirement for criterion b). These experts were recommended, if available, to gather a second PL expert in their country (purposeful sampling) to enable a four-eye principle for the provision of country information. In parallel, three members of the study (JC, KS, PE) theoretically derived curricular PL indicators supported by three members outside this core team (HG, SL, IP). The final set of indicators was implemented into an online survey (Webropol Survey v3·0, Helsinki, Finland). In the second stage, the core team (JC, KS), which did not participate in the survey, held two meetings with the experts to introduce and cooperatively organise the survey. The core team communicated the goal of this study to examine PL in the macropolitical context but did not mention the concrete country-level predictors a priori to avoid desirability bias. In the third stage, the country experts responded to the questions expressing the degree of physical education curricula's alignment with PL across primary education (5/6 to 10–12 years old), lower secondary education (11/12 to 14/15 years old), and upper secondary education (15/16 to 17/18 years old). Supplementary Material 2 describes how the experts dealt with the curricula that were not centrally organised at the national level. In the fourth stage, the first author (JC) analysed all quantitative PL questions, while another study member (KS) independently verified the statistical procedures. In line with the collaborative approach, the core team invited all experts to a joint discussion of the findings during the fifth stage, retrospectively informing them about the country-specific predictors underpinning the observed associations. Further details regarding the study design and process can be accessed in Carl and colleagues.^36^ This study followed the ‘Strengthening the Reporting of Observational Studies in Epidemiology’ (STROBE) Statement (Supplementary Material 3).

### Data sources

Curricular PL alignment was operationalised via 15 items with fixed anchor statements at both ends of a 5-point continuum.^36^ None of the items included the term ‘physical literacy’, reflecting the view that pedagogical practices can embody PL principles without explicit reference to the term. All items were derived from PL literature and iteratively developed within the core team through six internal feedback cycles and external advisors. To support completion of the survey, experts were provided with an offline version, enabling them to consult national documents on primary and secondary education, and make preparatory notes before submitting their responses via the online form. We reverse coded all negatively framed items and summed the responses to all 15 items to generate a composite score of ‘curricular PL alignment’. Initial psychometric analyses supporting the use of this theoretically derived scale (Cronbach's *α* = 0·86) can be accessed in Carl and colleagues.^36^ Supplementary Material 4 reports the results of a principal component analysis that adds factorial validity evidence to the scale, further corroborating the assumption that all items coherently represent ‘curricular PL alignment’ and that the findings of the scale can be interpreted in a unidimensional fashion. The scale ranged between 15 (minimum) to 75 (maximum) points.

To compute the planned associations, we retrieved country-level data from several international databases. Educational attainment was operationalised via the Organisation for Economic Co-operation and Development (OECD)'s 2022 Programme for International Student Assessment (PISA) test.^40^ The PISA score assesses general literacy and numeracy of lower secondary school students (aged 15 y old) across three dimensions: science, mathematics, and reading. The test scores are metric, ranging between 351 (Kosovo) to 516 (Estonia) in the 2022 wave. We extracted United Nations Human Development Index (HDI) as an aggregate score of human development.^41^ The HDI covers the dimensions ‘long and healthy life’, ‘knowledge’, and ‘standard of living’, with the scores (2021 dataset) ranging from 0·770 (North Macedonia) to 0·962 (Switzerland). Relative economic strength was captured using the Gross Domestic Product (GDP) per capita, based on 2023 data from the United Nations Statistics Division. GDP per capita values, expressed in US dollars, ranged from 4737 USD (Ukraine) to 128,936 USD (Luxembourg).^42^ The degree of liberty within a country was operationalised via the 2023 Human Freedom Index (HFI), published by CATO Institute, which aggregates twelve indicators of individual freedom.^43^ In the study sample, HFI scores ranged between 5·63 (Türkiye) and 9·01 (Switzerland). We applied the United Kingdom's score (8·39) to all four constituent countries of Great Britain and assigned Greenland the value of Denmark (8·83) due to administrative alignment. Finally, we measured innovative spirit using the 2023 Global Innovation Index (GII), which reflects both innovation input (e.g., human capital, research, institutions) and outputs (e.g., knowledge and technology output, creative goods and services).^44^ As GII information was not available for Greenland and significant innovation differences from Denmark were assumed, we had missing data for one country. The score ranged between 26·70 (Kazakhstan) and 67·60 (Switzerland). We took the most recent data of all five predictors from the time point of survey closing on 26 January 2024.

### Statistical analysis and visualisation

Each country's score of ‘curricular PL alignment’ was analysed descriptively. While a previous publication presented aggregated values across four European regions (plus the sum score in Supplementary Material),^36^ this current article provides detailed, individual item-level results. Crucially, we emphasised data visualisation by displaying all country scores using individual colour codes on a European map, which was generated via the open-source platform MapChart (goal 1). To enhance clarity and comparability, we transformed the unscaled sum score into a more interpretable percentage score, where 0% represented no curricular PL alignment and 100% full curricular PL alignment. The transformation (mapping) of scale scores and percentage values into corresponding metric colour was based on linear transitions within the RGB colour model, described in Supplementary Material 5. We checked via bivariate correlations (*r*) whether ‘curricular PL alignment’ depended on the time point since the last curricula reforms (separately for primary education, lower secondary education, and upper secondary education).

We calculated separate linear regression models for curricular PL alignment in relation to the five country-level predictors (goal 2): educational attainment (2022 PISA score), human development (2021 HDI), relative economic strength (2023 GDP per capita), societal liberty (2023 HFI), and innovative spirit (2023 GII). Notably, we checked whether all countries could be treated as independent cases or whether data was regionally clustered. Model comparisons revealed that the modelling of geospatial dependency via spatial regression did not substantially improve the model fit, justifying the application of linear regression models (Supplementary Material 6). To examine the robustness of significant associations, we tested alternative operationalisations or time points for the predictors (Supplementary Material 7). Non-linear regression variants did not improve the model fit or yield different results (Supplementary Material 8). We extracted standardised regression coefficients (*β*) for the interpretation of the effect sizes^45^ and computed 95% confidence intervals (CI_95_) using a two-sided testing procedure. Power analyses conducted in G∗Power v3·1^46^ indicated that, given the number of countries included, the study was powered to detect significant associations *β* ≥ 0·31. Since the five country-level predictors are often highly correlated and represent interconnected systemic characteristics, a multivariable model attempting to isolate the unique, independent effect of each variable would be methodologically unsound and challenging to interpret.^47^ To identify European countries with the strongest potential for improved PL alignment (goal 3), we analysed each country's deviation from the modelled trajectory using regression residuals (*e*), calculated as the difference between the observed curricular PL alignment score (*y*) and the expected value predicted by the regression equation ($\hat{y}$). Positive residuals (*e* > 0) indicated greater PL alignment than predicted by the respective country-level variable, while negative residuals (*e* < 0) indicated lower-than-expected PL alignment and were of particular interest for this study. All analyses were conducted in Rv4·4·1 with a significance level *p* < 0·05. We generated scatter plots with linear trend lines to visualise significant associations. The research team collaboratively discussed the findings and their implications on 17 April 2024, via Zoom v5·17 (Zoom Video Communications, San José, United States).

### Ethics statement

The present project was organised in Finland and following the Finnish National Board on Research Integrity (TENK) guidelines, the present study with academics screening national documents (publicly available information) and translating aggregated country information into a survey did not require ethical approval.

### Role of the funding source

There was no funding source for this study.

## Results

### Overview

We identified PL experts in 42 European countries: Austria, Belgium, Bosnia-Herzegovina, Bulgaria, Croatia, Cyprus, the Czech Republic, Denmark, England, Estonia, Finland, France, Germany, Greece, Greenland, Hungary, Iceland, Ireland, Italy, Kazakhstan, Latvia, Lithuania, Luxembourg, Moldova, Montenegro, the Netherlands, North Macedonia, Northern Ireland, Norway, Poland, Portugal, Romania, Scotland, Serbia, Slovakia, Slovenia, Spain, Sweden, Switzerland, Türkiye, Ukraine, and Wales. The contacted experts from Bosnia-Herzegovina and Moldova withdrew from the process, resulting in a final involvement of 72 experts from 40 European countries (for the final number of experts per country, see Supplementary Material 9). The experts were evenly distributed in terms of gender: 36 (50%) female, and 36 (50%) male. Furthermore, the experts were on average 45·7 (SD 9·1 years) old and had 20·8 (SD 10·2) years of teaching experience. Furthermore, 54 experts (75·0%) classified themselves as having a pedagogical background, 8 (11·1%) as having a sport science or coaching background, 5 (6·9%) as having a health science background, and 5 (6·9%) with another background. Data regarding the race and ethnicity of the experts were not collected, as the study unit of analysis was national policy alignment rather than individual-level demographics.

### Mapping the curricular alignment of European physical education with physical literacy (goal 1)

**Fig. 1:**
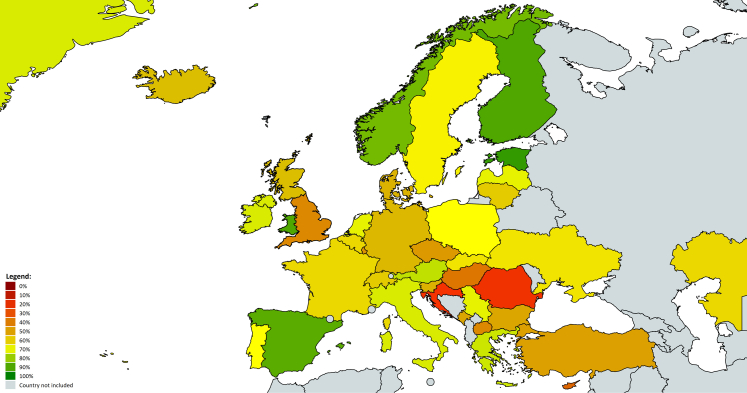
The degree of curricular physical literacy alignment across Europe. Note: Countries shown in green had higher alignment, while those in red showed lower alignment; for the entire algorithm transforming the physical literacy scores into visual representations, Supplementary Material 5.

The individual-country data for both the single items and the percentage scores can be found in Table 1. Estonia (93·3%), Wales (90·0%), Finland (90·0%), Spain (88·3%), and Norway (85·0%) displayed the highest values of curricular PL alignment, whereas Romania (23·3%), Croatia (25·0%), Cyprus (33·3%), England (43·3%), and North Macedonia (43·3%) the lowest. Fig. 1 visualises the degree of curricular PL alignment across Europe. The degree of curricular PL alignment was not significantly related to the time point since the latest curricula reforms in primary education (*r* = 0·14, *p* = 0·38), lower secondary education (*r* = 0·12, *p* = 0·48), and upper secondary education (*r* = −0·11, *p* = 0·51).

**Table 1:** Detailed overview of the country-specific ratings for PL-compatible pedagogy.

Country	Percentage score	Total score	i	ii	iii	iv	v	vi	vii	viii	ix	x	xi	xii	xiii	xiv	xv
Austria	75·0	60·0	4·0	5·0	4·0	3·0	5·0	4·0	3·0	5·0	1·0	5·0	3·0	5·0	5·0	4·0	4·0
Belgium	61·7	52·0	3·0	3·0	3·0	4·0	4·0	3·0	4·0	4·0	2·0	3·0	5·0	4·0	3·0	4·0	3·0
Bulgaria	51·7	46·0	1·0	3·0	1·0	4·0	4·0	3·0	5·0	4·0	5·0	3·0	2·0	5·0	3·0	2·0	1·0
Croatia	25·0	30·0	1·0	2·0	1·0	1·0	2·0	1·0	1·0	5·0	1·0	1·0	5·0	1·0	2·0	2·0	4·0
Cyprus	33·3	35·0	3·0	2·0	2·0	4·0	2·0	2·0	3·0	4·0	3·0	2·0	2·0	1·0	3·0	1·0	1·0
Czech Republic	48·3	44·0	3·0	2·0	3·0	4·0	4·0	2·0	2·0	3·0	2·0	4·0	3·0	3·0	4·0	3·0	2·0
Denmark	55·0	48·0	2·0	3·0	4·0	4·0	3·0	3·0	3·0	4·0	1·0	2·0	5·0	3·0	4·0	4·0	3·0
England	43·3	41·0	3·0	4·0	3·0	2·0	3·0	3·0	3·0	4·0	1·0	2·0	2·0	3·0	3·0	3·0	2·0
Estonia	93·3	71·0	5·0	5·0	4·0	5·0	5·0	5·0	5·0	5·0	4·0	4·0	5·0	4·0	5·0	5·0	5·0
Finland	90·0	69·0	5·0	5·0	5·0	4·0	5·0	5·0	5·0	5·0	2·0	3·0	5·0	5·0	5·0	5·0	5·0
France	61·7	52·0	2·0	2·0	2·0	4·0	5·0	2·0	4·0	4·0	4·0	4·0	2·0	4·0	5·0	4·0	4·0
Germany	56·7	49·0	3·0	5·0	3·0	3·0	2·0	3·0	3·0	4·0	2·0	3·0	4·0	3·0	4·0	4·0	3·0
Greece	73·3	59·0	4·0	4·0	4·0	5·0	4·0	3·0	5·0	5·0	1·0	4·0	4·0	2·0	5·0	5·0	4·0
Greenland	71·7	58·0	3·0	4·0	4·0	5·0	5·0	4·0	5·0	2·0	1·0	4·0	5·0	3·0	4·0	5·0	4·0
Hungary	38·3	38·0	1·0	3·0	4·0	2·0	4·0	2·0	3·0	4·0	1·0	2·0	2·0	4·0	3·0	2·0	1·0
Iceland	58·3	50·0	4·0	4·0	3·0	2·0	2·0	3·0	4·0	4·0	2·0	3·0	4·0	4·0	4·0	4·0	3·0
Ireland	71·2	57·7	2·0	4·0	4·0	5·0	3·0	4·0	4·0	4·0	2·0	4·0	4·0	3·7^a^	5·0	5·0	4·0
Italy	71·7	58·0	5·0	5·0	5·0	4·0	1·0	5·0	5·0	3·0	1·0	3·0	3·0	5·0	5·0	4·0	4·0
Kazakhstan	61·6	51·9	1·0	5·0	3·0	3·0	4·0	3·0	5·0	5·0	2·0	5·0	3·0	3·0	3·0	3·9^a^	3·0
Latvia	70·0	57·0	4·0	3·0	2·0	3·0	4·0	3·0	5·0	5·0	4·0	4·0	4·0	4·0	4·0	4·0	4·0
Lithuania	60·0	51·0	2·0	2·0	1·0	4·0	4·0	3·0	4·0	4·0	4·0	5·0	4·0	3·0	3·0	4·0	4·0
Luxembourg	46·7	43·0	2·0	3·0	2·0	4·0	3·0	2·0	3·0	4·0	1·0	3·0	2·0	4·0	3·0	2·0	5·0
Montenegro	51·7	46·0	4·0	2·0	4·0	4·0	2·0	2·0	4·0	4·0	2·0	3·0	3·0	3·0	4·0	3·0	2·0
Netherlands	70·0	57·0	4·0	4·0	4·0	3·0	4·0	4·0	4·0	4·0	3·0	3·0	4·0	4·0	4·0	4·0	4·0
North Macedonia	43·3	41·0	4·0	3·0	2·0	4·0	3·0	2·0	5·0	1·0	1·0	4·0	3·0	2·0	3·0	3·0	1·0
Northern Ireland	71·7	58·0	1·0	3·0	2·0	5·0	5·0	3·0	5·0	4·0	2·0	5·0	5·0	3·0	5·0	5·0	5·0
Norway	85·0	66·0	5·0	5·0	5·0	4·0	4·0	4·0	4·0	4·0	4·0	4·0	5·0	4·0	5·0	4·0	5·0
Poland	66·7	55·0	3·0	4·0	2·0	5·0	4·0	3·0	4·0	4·0	1·0	5·0	4·0	4·0	4·0	5·0	3·0
Portugal	66·7	55·0	3·0	4·0	4·0	3·0	5·0	3·0	5·0	5·0	3·0	4·0	1·0	5·0	5·0	2·0	3·0
Romania	23·3	29·0	1·0	2·0	3·0	1·0	1·0	1·0	2·0	2·0	1·0	2·0	4·0	2·0	3·0	2·0	2·0
Scotland	58·3	50·0	4·0	4·0	3·0	2·0	2·0	3·0	3·0	2·0	4·0	4·0	4·0	2·0	5·0	4·0	4·0
Serbia	70·0	57·0	1·0	3·0	1·0	3·0	5·0	3·0	5·0	5·0	4·0	5·0	4·0	4·0	4·0	5·0	5·0
Slovakia	63·3	53·0	3·0	4·0	3·0	4·0	4·0	4·0	3·0	4·0	4·0	2·0	2·0	5·0	4·0	3·0	4·0
Slovenia	55·0	48·0	4·0	4·0	3·0	2·0	4·0	3·0	3·0	3·0	1·0	3·0	2·0	4·0	5·0	4·0	3·0
Spain	88·3	68·0	5·0	5·0	3·0	4·0	5·0	4·0	5·0	5·0	2·0	5·0	5·0	5·0	5·0	5·0	5·0
Sweden	65·0	54·0	4·0	5·0	2·0	3·0	5·0	3·0	4·0	4·0	1·0	3·0	4·0	4·0	5·0	4·0	3·0
Switzerland	60·0	51·0	3·0	4·0	3·0	4·0	2·0	4·0	4·0	4·0	2·0	4·0	3·0	4·0	2·0	4·0	4·0
Türkiye	50·0	45·0	3·0	2·0	4·0	4·0	2·0	1·0	3·0	4·0	1·0	5·0	3·0	2·0	4·0	3·0	4·0
Ukraine	63·3	53·0	1·0	4·0	2·0	3·0	3·0	3·0	5·0	4·0	5·0	3·0	5·0	5·0	3·0	4·0	3·0
Wales	90·0	69·0	5·0	5·0	5·0	5·0	5·0	5·0	5·0	5·0	1·0	5·0	5·0	3·0	5·0	5·0	5·0

*Note:* The item values (range 1–5) are shown after inverting, if required. For the items, please see Supplementary Material 3.

The percentage values constitute a linear transformation of the sum score (15–60) and should not be interpreted in absolute numbers; small differences between countries should not be overstated.

a Two items (0·3%) were missing and were estimated using regression-based imputation.

### Country-level characteristics favouring curricular physical literacy alignment (goal 2)

**Fig. 2:**
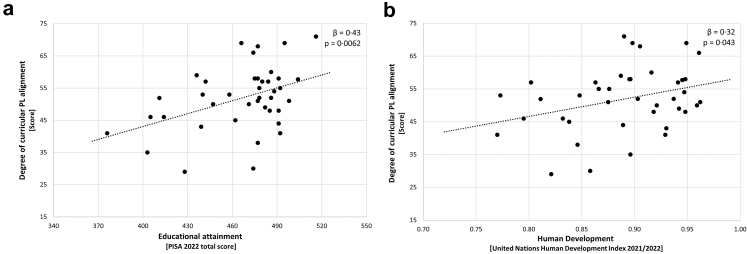
Country-level predictors of curricular physical literacy alignment. (a) The ‘educational attainment’ predictor; (b) the ‘human development’ predictor.

We identified a significant medium-to-large association across European countries between educational attainment and curricular PL alignment (*β* = 0·43 [CI_95_ 0·13, 0·65], *p* = 0·0062; Fig. 2a and Supplementary Material 7). Among the individual curricular PL alignment items, educational attainment was most strongly related to ‘encouragement of creativity and problem-solving’ (item xv; *β* = 0·47, *p* = 0·0025) and ‘student-identified purpose for activities’ (item xiv; *β* = 0·46, *p* = 0·0035). In contrast, the weakest relationships were observed for ‘embodiment and integration of body and mind’ (item vii; *β* = 0·10, *p* = 0·54) and ‘availability of assessment/charting for individual progress’ (item ix; *β* = −0·082, *p* = 0·61) (Supplementary Material 10). In addition, we found a significant association of medium effect size between the degree of human development and curricular PL alignment across the 40 countries (*β* = 0·32 [CI_95_ 0·011, 0·58], *p* = 0·043; Fig. 2b and Supplementary Material 7). Specifically, the items ‘student-centred acting’ (item i; *β* = 0·44, *p* = 0·0043) and ‘student involvement and provision of movement choice’ (item vi; *β* = 0·42, *p* = 0·0073) were most strongly related to the human development index, while the items ‘availability of assessment/charting methods for individual progress’ (item ix; *β* = −0·25, *p* = 0·11) and ‘embodiment and integration of body and mind’ (item vii; *β* = −0·14, *p* = 0·39) were the least (Supplementary Material 10).

**Fig. 3:**
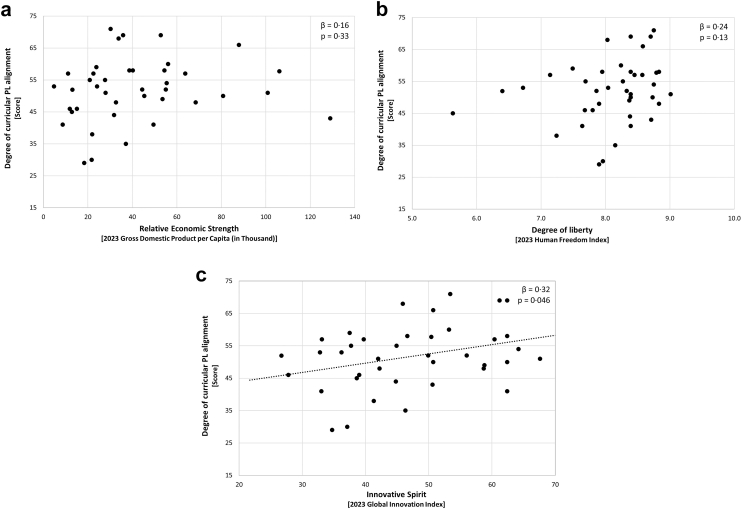
Country-level predictors of curricular physical literacy alignment. (a) The ‘relative economic strength’ predictor; (b) the ‘degree of liberty’ predictor; (c) the ‘innovative spirit’ predictor.

We did not record any association between relative economic strength and curricular PL alignment across European countries (*β* = 0·16 [CI_95_ −0·16, 0·45], *p* = 0·33; Fig. 3a), even after removing one notable outlier (Luxembourg; Supplementary Material 11). Similarly, the degree of liberty was not significantly related to curricular PL alignment across the 40 countries, *β* = 0·24 [CI_95_ −0·073, 0·52], *p* = 0·13 (Fig. 3b for a visualisation). In contrast, we registered a significant relationship of medium effect size between countries' innovative spirit and curricular PL alignment within Europe (*β* = 0·32 [CI_95_ 0·0071, 0·58], *p* = 0·046; Fig. 3c and Supplementary Material 7). Among the single PL items, innovative spirit was most strongly associated with the items ‘student involvement and provision of movement choice’ (item vi; *β* = 0·44, *p* = 0·0050) and ‘development of a meaningful relationship with physical activities’ (item ii; *β* = 0·37, *p* = 0·022), while the items ‘embodiment and integration of body and mind’ (item vii; *β* = −0·030, *p* = 0·86) and ‘availability of assessment/charting for individual progress’ (item ix; *β* = −0·11, *p* = 0·52) were the least (Supplementary Material 10).

### Identification of “laggards” in the curricular alignment of physical literacy (goal 3)

**Fig. 4:**
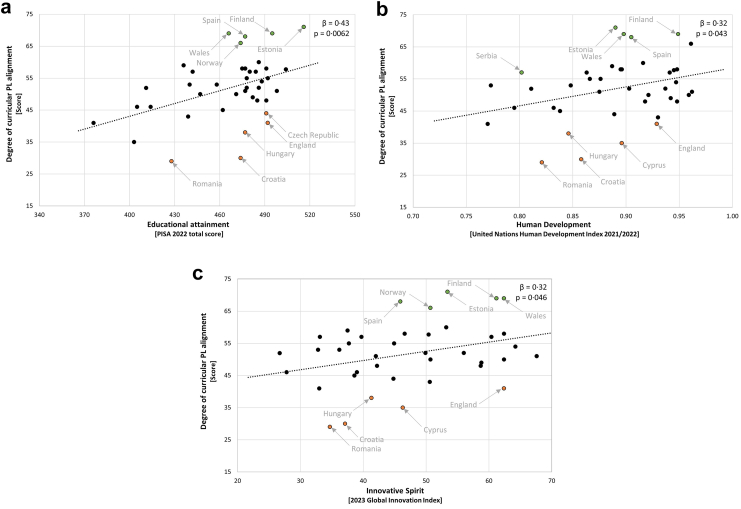
Identification of curricular ‘alignment laggards’ using regression residuals. (a) The significant ‘educational attainment’ predictor; (b) the significant ‘human development’ predictor; (c) the significant ‘innovative spirit’ predictor.

Taking into account the significant association between countries’ educational attainment and curricular PL alignment, the values of Croatia (*e* = −22·9), Romania (*e* = −17·8), Hungary (*e* = −15·3), England (*e* = −14·3), and the Czech Republic (*e* = −11·2) demonstrated the strongest negative deviations from the modelled regression line (Fig. 4a and Supplementary Material 12). Conversely, Wales (*e* = 17·1), Spain (*e* = 14·7), Finland (*e* = 13·3), Norway (*e* = 13·1), and Estonia (*e* = 12·5) exhibited the strongest positive deviations from the regression line.

In relation to human development, the values of PL alignment from Croatia (*e* = −20·0), Romania (*e* = −18·9), Cyprus (*e* = −17·3), England (*e* = −13·2), and Hungary (*e* = −11·3) showed the largest negative deviations from the regression line (Fig. 4b and Supplementary Material 12). Positive deviations from the regression line were strongest for Estonia (*e* = 19·9), Wales (*e* = 16·6), Spain (*e* = 15·2), Finland (*e* = 13·6), and Serbia (*e* = 10·3).

With respect to innovative spirit and curricular PL alignment, Romania (*e* = −19·1), Croatia (*e* = −18·8), Cyprus (*e* = −16·5), England (*e* = −15·1), and Hungary (*e* = −12·0) exhibited the largest negative deviations from the regression line (Fig. 4c and Supplementary Material 12). The most pronounced positive deviations were again observed for Estonia (*e* = 17·5), Spain (*e* = 16·7), Norway (*e* = 13·3), Finland (*e* = 13·3), and Wales (*e* = 12·9).

## Discussion

Physical education plays a pivotal role in cultivating lifelong engagement in health-enhancing physical activity, with the promotion of PL having gained significant traction on the global agendas of health and education policy.^1^^,^^6^^,^^7^ Recognising the emphasis of this integrative concept for individual development and physical activity, and despite the existence of varied conceptualisations, this study extended beyond individual contexts to broadly examine the relevance of PL within physical education curricula across Europe in three different objectives.

The present study investigated whether the different European countries effectively incorporated PL principles into their physical education curricula (first objective). This knowledge is essential to investigate the contribution of formalised physical education to quality education and sustained physical activity for health through PL across the continent.^1^^,^^6^^,^^7^ We identified substantial heterogeneity within Europe regarding the formal alignment of physical education with the holistic PL concept. In summary, the physical education curricula across the 40 countries only moderately met UNESCO's and WHO's claims to align policies and curricula with PL. The presented map might serve as an important visualisation tool for health and education policy to proactively support the development of PL-compatible curricula in the future.^48^ While the literature has revealed several studies that geographically map quantitative levels of physical activity behaviour,^49^^,^^50^ such visual tools are still largely missing for health and education policy. Leveraging the illustrative potential of colour, in particular the darker countries might be inspired to successively incorporate aspects of PL into future curriculum reforms for physical education. Although the items (due to the study location) may have been most strongly inspired by the understanding of the International Physical Literacy Association, the PL indicators were developed to remain independent of debates about the definition of PL, prioritising relevance across diverse interpretations. Nonetheless, the idea of a fully ‘ideal’ PL-aligned curriculum requires caution. Complete alignment may be difficult to achieve in practice, given national mandates, cultural traditions, and structural constraints. Maximal PL alignment should, therefore, be viewed as a direction of continuous improvement rather than a fixed or prescriptive endpoint. The 15 items operationalising curricular PL alignment exemplified the spectrum of how educational goals and principles may successively shift towards a stronger focus on PL. For instance, physical education curricula could more strongly emphasise creativity and problem-solving instead of focusing on the internalisation of movement patterns (see the indicative item xv); or curriculum reforms could include a shift away from teacher-centred instruction to stronger student participation and reflections about own preferences (see the indicative item vi).

Building upon this argument favouring a stronger health and education policy in line with PL, the present study found that the alignment of physical education curricula with PL follows educational attainment, human development, and innovation gradients (second objective). Transcending the line of development from theoretical studies to individual-level evidence within research on PL, this is, to our knowledge, the first study to demonstrate such associations at the country level. The integration of PL-driven principles into the curricula of physical education might reflect certain features of educational systems, such as stronger focus on holistic learning and long-term personal development, uncertainty tolerance and better openness to international discussions, more frequent curriculum reforms, and an appreciation of creative solutions (e.g., divergent thinking).^37^^,^^51^^,^^52^ Supported by strong empirical interrelations, the combination of educational attainment, focus on human development, and innovative spirit may form a cluster that is positively associated with the adoption of PL within physical education. The non-significant results with relative economic strength suggest that the alignment of curricula in physical education with PL might be less directly dependent on the economic situation of a country. Assuming that the adoption of PL might reflect a country's general tendency to place the individual in the centre of educational endeavours,^53^ we also examined associations with an indicator of human freedom but the findings did not confirm this assumption. At this point, we aim to consider that, in line with the observational nature of the present data, the identified relationships may partially arise from reverse influences. Countries' human-centred worldview, culminating in consideration of PL within physical education curricula, might be associated with higher standards of education, human development, and innovation. Theoretical articles have argued that PL can lead to better education, human flourishing, positive youth development, health, well-being, and even innovation knowledge.^8^^,^^9^^,^^24^^,^^26^^,^^54^ Empirical studies in recent years have accumulated increasing evidence that children's PL has a positive effect on cognition and academic performance^55^^,^^56^ as well as on health and quality of life.^27^^,^^28^ However, researchers should recognise the limited time spent in physical education at school (relative to all other influences that occur through a person's life) when attempting to attribute the medium-to-large effect sizes (0·32 ≤ *β* ≤ 0·43) to reverse associations between physical education and children's education, personal development, and innovative capacity. It is highly plausible that unexamined third variables or complex systemic factors simultaneously drive both higher educational attainment and greater curricular flexibility towards concepts like PL. Nevertheless, the alternative direction in the explanation would not deliver an argument against a stronger emphasis of PL in physical education.

The final analysis explicitly considered educational attainment, human development, and innovation gradients to gather insights about relative ‘laggards’ and ‘pioneers’ (third objective). Interpreting this study as a descriptive overview, we aim to emphasise the exploratory nature of the residual analysis. Further qualitative and contextual work is required before drawing strong conclusions or policy mandates. Estonia, Spain, Finland, and Wales consistently surpassed the expected degree of PL alignment given their relative country conditions (*e* > 0) and might, therefore, be positioned as ‘pioneers’ for implementing PL into physical education curricula. In contrast, the following countries congruently scored lower than predicted by their levels of educational attainment, human development, and innovative spirit (*e* < 0): Romania, Croatia, Cyprus, England, Hungary, Luxembourg, Czech Republic, Denmark, Switzerland, Scotland, Germany, Türkiye, Sweden, Slovenia, France, Belgium, and Montenegro. Most importantly, Croatia, Romania, Hungary, and England consistently ranked among the five countries with the strongest negative deviation. Supported by contrasting colours in the initial map, Wales and England scored differently in curricular PL alignment despite their common cultural and linguistic roots in Great Britain. While the curriculum in Wales has undergone its latest reform in 2023,^57^ England as the originating place of PL has not completed any curricular change since 2014 at the timepoint of data collection.^58^ This circumstance may have become visible in the present examination. Although the Nordic countries scored well overall, some differences became visible. For instance, the Finnish education system strongly supports healthy lifestyles attempting to reduce sportive elements with the latest curriculum reform, while the physical education curricula in Sweden have more strongly prioritised all-round movement competence without a clear holistic perspective on bodily learning and healthy lifestyle habits.

Supported by a recent study from the United States demonstrating that the effect of quality physical education on physical activity is significantly explained by targeting children's PL,^16^ we assume that the present findings, including the European mapping, the identification of country-level correlates, and the associative residual errors, have crucial relevance for health and education policy. Theoretical work and recent individual-level studies suggest that a stronger adoption of PL in physical education is a plausible upstream determinant with the potential to support diverse developmental outcomes and to foster physical activity for biopsychosocial health.^8^^,^^17^^,^^18^^,^^24^^,^^25^ However, we must emphasise that making inferences about individual-level benefits based on systems-level findings and potential changes (e.g., in curricula) risk the ecological fallacy.^59^ Despite the implications from this study and the prominent emphasis of PL propelled by UNESCO's and WHO's health and education policy, stakeholders should recognise that educational reforms constitute a complex, politicised, and culturally situated process, which–if inadequately organised–carries the risk of provoking reactive behaviour towards change.^60^^,^^61^ Instead of externally forcing certain developments, curricular reform should adhere to participatory principles, giving decision-makers the perception that the planned change resonates with intrinsic values and the individual political agenda. Against this background, we recommend using the descriptive map and findings for open discussion with care and without pressure when presenting to national decision-makers. Importantly, while PL has found strong political support at the global level, the academic landscape continues to critically discuss PL.^62^^,^^63^ In addition to the diversity in definitions as mentioned above,^11^ researchers have pointed to philosophical tensions^32^^,^^64^ as well as competing traditions of thought that shape its meaning and scholarly practices (e.g., measurement).^65^, ^66^, ^67^ Therefore, it is crucial to explain whether PL is used in a political-normative, descriptive-analytical, or a critical context.

This study incorporated some methodological strengths, such as the theoretically guided development of items to assess curricular PL alignment, the inclusion of many European countries, the examination of geospatial dependency, the robustness analyses, the exploration of non-linear associations, and the consideration of a visual outlier. However, we discuss the following limitations for this study. First, the information about curricular PL alignment relied on expert-reported data. Aware of potential biases resulting from reporting health and education policies, we (a) enabled the experts to undertake searches for obtaining necessary information (e.g., for different curricula at the sub-national level) by sending an offline variant of the items at the beginning of the survey phase; (b) provided statements at both ends of the scale for better orientation; (c) deliberately varied the location of the PL-compatible statement (left versus right) across the survey; (d) renounced visible information about the PL-compatible statement in the survey; and (e) undertook psychometric examinations (at both item and scale levels) within the constraints imposed by the sample size. In this context, we also advised the experts, whenever possible, to work in pairs. However, eight countries were only represented by one expert. These countries were significantly smaller, had lower educational attainment, and had lower innovative spirit relative to countries with two experts (see Supplementary Material 9). Instead of removing these countries from the analyses, we considered it essential to include these for continental representativeness as well as developmental purposes. In summary, the expert approach, albeit tested psychometrically, was susceptible to inconsistent interpretations of PL principles and varied understandings of survey items across cultures. Future studies could complement the subjective rating through objective coding of curriculum texts. Second, we again mention the observational nature of the data and bidirectional relationship between the country-level predictors and PL alignment. Acknowledging the difficulty in health and education policy studies to employ experimental designs or causal analyses,^68^ stakeholders are cautioned against interpreting the identified statistical associations as evidence of causality. Future research should prioritize longitudinal designs to further illuminate the complex interdependencies behind these country-level determinants. This study marked a first attempt to approach this relationship through an observational design using recognised country-level indices across the European continent. Third, this study examined European physical education curricula from a health and education policy perspective, thereby focusing on the formal level of physical education curricula. While an investigation into the enacted curriculum (i.e., how curricula are effectively implemented in practice) fell outside the scope of this study, such data would have offered crucial complementary insights into the challenges of curriculum design. Finally, we could not involve PL experts from all European countries, as we could not identify any PL experts for some smaller and Eastern European countries, or the political situation undermined some cooperation. A successive inclusion of more countries would not only deliver a more representative picture of the continental situation but also increase the sample size, reduce the beta error masking relevant associations, and enhance the variance to detect associations in the future.

While the concept of PL has found its way onto the global agenda of health and education, this study demonstrated that the European countries have only hesitantly integrated its principles into national curricula. In summary, the analyses revealed large heterogeneity in the compatibility of physical education curricula with PL across Europe. The identified associations of educational attainment, human development, and innovative spirit with curricular PL alignment suggested the first country-level correlates for enhanced incorporation of PL principles into the physical education system, inviting stakeholders in research, practice, and policy to consider these findings when enriching and adjusting existing policies, programmes, and strategies. The literature-derived items uncovered a spectrum between PL-incompatible and PL-compatible curricula, offering guidance on how goals, instructions, and outcomes in physical education contexts might be thoroughly shifted towards stronger alignment with PL. Ultimately, these findings invite a targeted policy focus—particularly in ‘alignment laggard’ countries—to harness the potential of physical literacy for lifelong physical activity and public health through student-centred education.

## Contributors

Conceptualisation: Johannes Carl, Kasper Salin, Petr Vlček, Cristiana D'Anna & Peter Elsborg; Country-specific reviews and tables: All authors, including the consortium authors, but except of the first, second, and third authors; Data curation: Kasper Salin; Formal analysis (primary calculations) with access to the raw data: Johannes Carl; Formal analysis (data verification) with access to the raw data: Kasper Salin; Funding acquisition: no specific funding; Investigation: Johannes Carl & Kasper Salin; Methodology (primary development): Johannes Carl & Kasper Salin; Methodology (feedback): Hannah Goss, Suzanne Lundvall & Iulia Pavlova; Project administration: Johannes Carl & Kasper Salin; Software: Johannes Carl & Kasper Salin; Supervision: Lisa Barnett, Petr Vlček & Cristiana D'Anna; Revalidation: All authors, except of the first, second, and third authors; Visualisation: Johannes Carl; Writing–original draft: Johannes Carl; Writing–review & editing: All authors. More than one author has verified the data (Johannes Carl & Kasper Salin). The first (and corresponding) author has the final responsibility for the decision to submit for publication. All authors, including the consortium authors, have read and approved the final version of the manuscript.

## Data sharing statement

Country-level data (no participant information) will be made available with publication in Open Science Framework (OSF) under the following Digital Object Identifier (doi): 10.17605/OSF.IO/6MPQ8.

## Editor note

The Lancet Group takes a neutral position with respect to territorial claims in published maps and institutional affiliations.

## Declaration of interests

The authors declare that they have no known competing financial interests or personal relationships that could have appeared to influence the work reported in this paper.
