# Supplementary material for: Alignment of physical education curricula with physical literacy across Europe: an observational mapping study with country-level predictors
Source: Lancet Reg Health Eur. 2026 Mar 10;65:101641. doi: 10.1016/j.lanepe.2026.101641 (PMC12994078; doi:10.1016/j.lanepe.2026.101641)
Supplement: Supplementary File [file mmc1.docx]

Supplementary Material

## Supplementary Material 1. Background explanation to the selection of experts for the formation of the EUROPLIT network (study phase 1).

The International Physical Literacy Organisation (IPLA) constitutes a non-governmental organisation at the international scale which promotes and monitors exchange on matters of PL by developing initiatives on research, advocacy, and education. Following discussions at the 2021 annual conference of the IPLA, the first author (JC) contacted the board (NG) of the society to identify potential experts (e.g., persons who actively delved with the concept or had topic-related publications) for PL in Central Europe. After candidates were appointed for five countries (Austria, the Czech Republic, Denmark, France, and Switzerland), individual contacts were established via electronic mail and the experts were invited to join the present initiative. As a result of initial conversations, the experts from two additional countries were recruited through snowballing principle (Germany, Belgium) and the group agreed in a joint meeting to transcend the narrow Central Europe perspective by involving experts from other regions of Europe as well. This step finally led to experts from Croatia, Cyprus, England, Finland, Greece, Italy, Lithuania, the Netherlands, Norway, Poland, Portugal, Romania, Scotland, Wales, Spain, Sweden, Türkiye, and Ukraine. Despite encompassing a total of 25 countries, the first study possessed the weakness that not all European countries were covered. In particular, smaller countries and Eastern European countries were missing disproportionately (Carl et al., 2023), implying that it would be beneficial for future projects to enhance representativity across the continent. Accordingly, we invested increasing efforts prior to this second academic endeavour in finding experts in these countries by specifically making contacts at academic conferences (one specifically in Eastern Europe). In this context, the effort was successful in additionally including experts from the following countries: Bulgaria, Estonia, Greenland, Hungary, Iceland, Ireland, Kazakhstan, Latvia, Luxembourg, Montenegro, North Macedonia, Northern Ireland, Serbia, Slovakia, Slovenia. Experts from Bosnia-Herzegovina and Moldova were invited to participate but withdrew from the process.

## Supplementary Material 2. Information regarding the organisation of physical education curricula within Europe, with subsequent explanations for the reporting at sub-national levels (if necessary).

| **Country** | **Level of Organisation for the Physical Education Curricula** | **Explanation Regarding the Experts’ Assessment for  Countries with Multiple Curriculum Sources^1^** |
| --- | --- | --- |
| Austria | National Level | - |
| Belgium | Sub-national level | Belgium has three Community-level PE curricula. The ratings combine the Flemish (60%) and French (39%) curricula, covering 99% of pupils; the German-speaking curriculum (1%) was excluded because of its very small population share and low representativeness. The Flemish and French Community experts systematically compared how each item was embedded in their respective curricula and, where necessary, explicitly discussed inter-Community differences. Based on this comparative appraisal, an internal aggregated value was jointly derived to represent the curricular situation across the two major Communities. |
| Bulgaria | National Level | - |
| Croatia | National Level | - |
| Cyprus | National Level | - |
| Czech Republic | National Level | - |
| Denmark | National Level | - |
| England | State Level | - |
| Estonia | National Level | - |
| Finland | National Level | - |
| France | National Level | - |
| Germany | Sub-national level | 15 different curricula: the experts referred to the curricula of four federal states in Germany: Baden-Württemberg, North Rhine Westphalia, Saxonia, and Thuringia. They represent curricula from former East-Germany and West-Germany. Crucially, North Rhine Westphalia as Germany’s most populous state (~16M) is a typical representative within Germany’s education landscape. |
| Greece | National Level | - |
| Greenland | Regional Level | - |
| Hungary | National Level | - |
| Iceland· | National Level | - |
| Ireland | National Level | - |
| Italy | National Level | - |
| Kazakhstan | National Level | - |
| Latvia | National Level | - |
| Lithuania | National Level | - |
| Luxembourg | National Level | - |
| Montenegro | National Level | - |
| Netherlands | National Level | - |
| North Macedonia | National Level | - |
| Northern Ireland | State Level | - |
| Norway | National Level | - |
| Poland | National Level | - |
| Portugal | National Level | - |
| Romania | National Level | - |
| Scotland | State Level | - |
| Serbia | National Level | - |
| Slovakia | National Level | - |
| Slovenia | National Level | - |
| Spain | Sub-national level | The educational curriculum in Spain is organised through a common national framework, defined by the Ministry of Education through organic laws and royal decrees that establish the compulsory minimum standards for the entire country. Based on this shared framework, the autonomous regions further develop and complete the curriculum at the regional level, adapting it to their social, cultural, and linguistic contexts and incorporating their own specific content. In this way, while the national level ensures the basic homogeneity and equivalence of the education system, the regions retain a degree of autonomy to introduce distinct educational approaches, priorities, and specific developments without undermining the unity of the system. Therefore, the data were mainly drawn from the national framework, which determines the compulsory minimum teaching requirements. |
| Sweden | National Level | - |
| Switzerland | Sub-national level | There are three different curricula in Switzerland (Curriculum 21 [German-speaking Switzerland], Plan d'études romand [PER] [French-speaking Switzerland] and Piano di studio [Italian-speaking Ticino]). The two experts filled in the information for German-speaking Switzerland, representing 21 cantons, ~ 5.9M or 66% of the Swiss population. Previous curriculum analyses have shown that this intercantonal, regional curriculum is representative of the Swiss educational landscape. |
| Türkiye | National level | - |
| Ukraine | National Level | - |
| Wales | State Level | - |

## Note: Excluding differentiations by school level (e.g., primary versus secondary education). Status of the curricula from January 2024.

## Supplementary Material 3. STROBE Statement—checklist of items for inclusion in observational studies.

|  | **Item No** | **Recommendation** | **Page Number (Main File)** |
| --- | --- | --- | --- |
| **Title and abstract** | 1 | (*a*) Indicate the study’s design with a commonly used term in the title or the  Abstract | 1 |
|  |  | (*b*) Provide in the abstract an informative and balanced summary of what was done and what was found | 1/2 |
| **Introduction** | | | |
| Background/rationale | 2 | Explain the scientific background and rationale for the investigation being  reported | 3-4 |
| Objectives | 3 | State specific objectives, including any prespecified hypotheses | 4/5 |
| **Methods** | | | |
| Study design | 4 | Present key elements of study design early in the paper | 5-6 |
| Setting | 5 | Describe the setting, locations, and relevant dates, including periods of recruitment, exposure, follow-up, and data collection | 5-6, Supplementary  Material 1 |
| Participants | 6 | (*a*) *Cohort study*—Give the eligibility criteria, and the sources and methods of selection of participants. Describe methods of follow-up  *Case-control study*—Give the eligibility criteria, and the sources and methods of case ascertainment and control selection. Give the rationale for the choice of cases and controls  *Cross-sectional study*—Give the eligibility criteria, and the sources and methods  of selection of participants | 5-6, Supplementary Material 1, and reference to validation article |
|  |  | (*b*) *Cohort study*—For matched studies, give matching criteria and number of exposed and unexposed  *Case-control study*—For matched studies, give matching criteria and the number  of controls per case | This is not a matched study |
| Variables | 7 | Clearly define all outcomes, exposures, predictors, potential confounders, and effect modifiers. Give diagnostic criteria, if applicable | 4-5 and 6-8 |
| Data sources/ measurement | 8* | For each variable of interest, give sources of data and details of methods of assessment (measurement). Describe comparability of assessment methods if  there is more than one group | 6-7 |
| Bias | 9 | Describe any efforts to address potential sources of bias | 8, Supplementary  Material 6, 7 and 8 |
| Study size | 10 | Explain how the study size was arrived at | 5 and 9, Supplementary  Material 1 and 9 |
| Quantitative variables | 11 | Explain how quantitative variables were handled in the analyses. If applicable, describe which groupings were chosen and why | 8-9 |
| Statistical methods | 12 | (*a*) Describe all statistical methods, including those used to control for  confounding | 8,97 |
|  |  | (*b*) Describe any methods used to examine subgroups and interactions | Not relevant, Supplementary Material  11 for outlier |
|  |  | (*c*) Explain how missing data were addressed | 7, Table 1 |
|  |  | (*d*) *Cohort study*—If applicable, explain how loss to follow-up was addressed  *Case-control study*—If applicable, explain how matching of cases and controls was addressed | 8, 9 |

1

|  | |  | *Cross-sectional study*—If applicable, describe analytical methods taking account of sampling strategy |  |
| --- | --- | --- | --- | --- |
|  |  |  | (*e*) Describe any sensitivity analyses | Supplementary Material  11 |
| **Results** | | | | |
| Participants | 13* | (a) Report numbers of individuals at each stage of study—eg numbers potentially eligible, examined for eligibility, confirmed eligible, included in the study, completing follow-up, and analysed | | 9 and 10, Supplementary File 1 |
|  |  | (b) Give reasons for non-participation at each stage | | Supplementary File 1 |
|  |  | (c) Consider use of a flow diagram | | We considered it but  finally did not include it |
| Descriptive data | 14* | (a) Give characteristics of study participants (e.g., demographic, clinical, social) and information on exposures and potential confounders | | 9-10 |
|  |  | (b) Indicate number of participants with missing data for each variable of interest | | Table 1 |
|  |  | (c) *Cohort study*—Summarise follow-up time (e.g., average and total amount) | | This is not a cohort study |
| Outcome data | 15* | *Cohort study*—Report numbers of outcome events or summary measures over time | | This is not a cohort study |
|  |  | *Case-control study—*Report numbers in each exposure category, or summary measures of exposure | | This is not a case-control study |
|  |  | *Cross-sectional study—*Report numbers of outcome events or summary measures | | 8 |
| Main results | 16 | (*a*) Give unadjusted estimates and, if applicable, confounder-adjusted estimates and their precision (eg, 95% confidence interval). Make clear which confounders were adjusted for  and why they were included | | 10-12 |
|  |  | (*b*) Report category boundaries when continuous variables were categorised | | 10-11 |
|  |  | (*c*) If relevant, consider translating estimates of relative risk into absolute risk for a meaningful time period | | Not relevant for this study |
| Other analyses | 17 | Report other analyses done—eg analyses of subgroups and interactions, and sensitivity analyses | | 10-12, including reference to extensive Supplementary Material |
| **Discussion** | | | | |
| Key results | 18 | Summarise key results with reference to study objectives | | 12-15 |
| Limitations | 19 | Discuss limitations of the study, taking into account sources of potential bias or  imprecision. Discuss both direction and magnitude of any potential bias | | 16-17 |
| Interpretation | 20 | Give a cautious overall interpretation of results considering objectives, limitations, multiplicity of analyses, results from similar studies, and other relevant evidence | | 12-17 |
| Generalisability | 21 | Discuss the generalisability (external validity) of the study results | | 14-17 |
| **Other information** | | | | |
| Funding | 22 | Give the source of funding and the role of the funders for the present study and, if applicable, for the original study on which the present article is based | | 18 |

*Give information separately for cases and controls in case-control studies and, if applicable, for exposed and unexposed groups in cohort and cross-sectional studies.

2

**Supplementary Material 4. Principal component analysis with (a) the factor loadings and (b) an exploratory scree plot.**

**
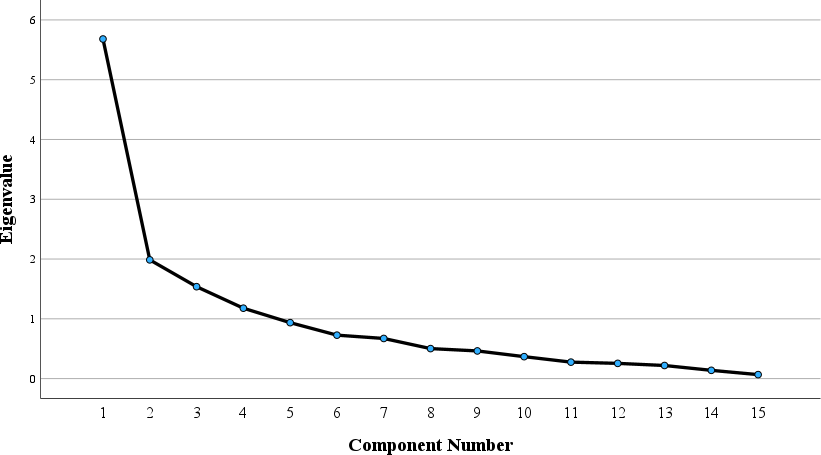
**

| **Item**  **Number** | **Physical literacy aspect** | **Factor  loadings** |
| --- | --- | --- |
| i | Student-centred acting | 0·58 |
| ii | Development of a meaningful relationship with physical activities | 0·74 |
| iii | Acknowledgment of ‘unique journeys’ | 0·48 |
| iv | Balance between physical, cognitive, and psychosocial aspects | 0·57 |
| v | Exploration of different activity contexts | 0·61 |
| vi | Student involvement and the provision of movement choice | 0·86 |
| vii | Embodiment and the integration of body and mind | 0·69 |
| viii | Building own responsibility for engagement in physical activities | 0·38 |
| ix | Availability of assessment/charting methods for individual progress | 0·13 |
| x | Transcendence of the current horizon through ‘lifelong learning’ | 0·59 |
| xi | Prioritisation of variety and exploration | 0·43 |
| xii | Reward and participation for everyone | 0·54 |
| xiii | Inclusion and accessibility of PE for all | 0·72 |
| xiv | Student-identified purpose for activities | 0·83 |
| xv | Encouragement of creativity and problem-solving. | 0·69 |

Note: The factor loadings were retrieved from an unidimensional factor solution. The scree plot, generated within the scope of principal component analysis (PCA), only showed a single clear point of deflection, supporting the assumption that the 15 items can be interpreted against the backdrop of one dimension (called “curricular PL alignment”).

Please consider that we were limited in our ability to conduct psychometric analyses on the country level given the sample size (e.g., no confirmatory factor analysis possible; see limitation section).

**Supplementary Material 5. Transformation of the physical literacy scores into visual representations for the mapping using linear transitions with the RGB colour system.**

| **Score (Scale)** | **Percentage Score** | **Visual** | **Red** | **Green** | **Blue** |
| --- | --- | --- | --- | --- | --- |
| 15 | 0·0 |  | 140 | 0 | 0 |
| 16 | 1·7 |  | 148 | 4 | 0 |
| 17 | 3·3 |  | 156 | 8 | 0 |
| 18 | 5·0 |  | 164 | 12 | 0 |
| 19 | 6·7 |  | 172 | 16 | 0 |
| 20 | 8·3 |  | 180 | 20 | 0 |
| 21 | 10·0 |  | 188 | 25 | 0 |
| 22 | 11·7 |  | 196 | 30 | 0 |
| 23 | 13·3 |  | 204 | 35 | 0 |
| 24 | 15·0 |  | 212 | 40 | 0 |
| 25 | 16·7 |  | 220 | 45 | 0 |
| 26 | 18·3 |  | 225 | 46 | 0 |
| 27 | 20·0 |  | 230 | 47 | 0 |
| 28 | 21·7 |  | 235 | 48 | 0 |
| 29 | 23·3 |  | 240 | 49 | 0 |
| 30 | 25·0 |  | 245 | 50 | 0 |
| 31 | 26·7 |  | 240 | 60 | 0 |
| 32 | 28·3 |  | 235 | 70 | 0 |
| 33 | 30·0 |  | 230 | 80 | 0 |
| 34 | 31·7 |  | 225 | 90 | 0 |
| 35 | 33·3 |  | 220 | 100 | 0 |
| 36 | 35·0 |  | 220 | 106 | 0 |
| 37 | 36·7 |  | 220 | 112 | 0 |
| 38 | 38·3 |  | 220 | 118 | 0 |
| 39 | 40·0 |  | 220 | 124 | 0 |
| 40 | 41·7 |  | 220 | 130 | 0 |
| 41 | 43·3 |  | 220 | 136 | 0 |
| 42 | 45·0 |  | 220 | 142 | 0 |
| 43 | 46·7 |  | 220 | 148 | 0 |
| 44 | 48·3 |  | 220 | 154 | 0 |
| 45 | 50·0 |  | 220 | 160 | 0 |
| 46 | 51·7 |  | 220 | 166 | 0 |
| 47 | 53·3 |  | 220 | 172 | 0 |
| 48 | 55·0 |  | 220 | 178 | 0 |
| 49 | 56·7 |  | 220 | 184 | 0 |
| 50 | 58·3 |  | 220 | 190 | 0 |
| 51 | 60·0 |  | 227 | 203 | 0 |
| 52 | 61·7 |  | 234 | 216 | 0 |
| 53 | 63·3 |  | 241 | 229 | 0 |
| 54 | 65·0 |  | 248 | 242 | 0 |
| 55 | 66·7 |  | 255 | 255 | 0 |
| 56 | 68·3 |  | 242·3 | 248·7 | 0 |
| 57 | 70·0 |  | 229·6 | 242·4 | 0 |
| 58 | 71·7 |  | 216·9 | 236·1 | 0 |
| 59 | 73·3 |  | 204·2 | 229·8 | 0 |
| 60 | 75·0 |  | 191·5 | 223·5 | 0 |
| 61 | 76·7 |  | 178·8 | 217·2 | 0 |
| 62 | 78·3 |  | 166·1 | 210·9 | 0 |
| 63 | 80·0 |  | 153·4 | 204·6 | 0 |
| 64 | 81·7 |  | 140·7 | 198·3 | 0 |
| 65 | 83·3 |  | 128 | 192 | 0 |
| 66 | 85·0 |  | 115·2 | 185·6 | 0 |
| 67 | 86·7 |  | 102·4 | 179·2 | 0 |
| 68 | 88·3 |  | 89·6 | 172·8 | 0 |
| 69 | 90·0 |  | 76·8 | 166·4 | 0 |
| 70 | 91·7 |  | 64 | 160 | 0 |
| 71 | 93·3 |  | 51·2 | 153·6 | 0 |
| 72 | 95·0 |  | 38·4 | 147·2 | 0 |
| 73 | 96·7 |  | 25·6 | 140·8 | 0 |
| 74 | 98·3 |  | 12·8 | 134·4 | 0 |
| 75 | 100·0 |  | 0 | 128 | 0 |

Note: Knots for the linear transitions (by scale scores): 20, 25, 30, 35, 50, 55, 65.

## Supplementary Material 6. Examination of assumptions of geospatial independence versus dependency.

The regression requires independent observations between the predictors and curricular PL alignment. To formally test this assumption, we conducted analyses for spatial independence (using Moran’s I test to detect spatial autocorrelations) and calculated spatial regression models using Spatial Lag Models (to detect spatial spillover effects) and Spatial Error Models (to check whether spatial dependency is captured within the error terms of the model).

The spatial location of each country was based on the geographical centroids of each country/region and the K-Nearest Neighbours approach. These coordinates served as the precise location points for all subsequent spatial analyses. A fixed number of nearest neighbours (K=5) was chosen, meaning that every country was spatially linked to its five closest geographical neighbours. The neighbourhood list was converted into a spatial weight matrix for further processing.

| **Predictors** | **Test for Spatial Autocorrelations** | | **Linear Regression  (without spatial dependency)** | | | | **Spatial Regression Models** | | | | **Model Comparison^1^ (Likelihood-Ratio Test)** | |
| --- | --- | --- | --- | --- | --- | --- | --- | --- | --- | --- | --- | --- |
|  | *Moran’s I* | *p* | *β* | *p* | *AIC* | *BIC* | Model Type | *ρ* | *AIC* | *BIC* | *∆χ²* | *p* |
| Educational attainment [PISA] | 0·023 | 0·553 | 0·426 | 0·006 | 110·51 | 115·57 | Spatial Lag Model | *ρ* = 0·153 | 112·06 | 118·81 | 0·449 | 0·503 |
|  |  |  |  |  |  |  | Spatial Error Model | *λ =* 0·069 | 112·44 | 119·20 | 0·065 | 0·799 |
| Human development  [HDI] | 0·067 | 0·265 | 0·322 | 0·043 | 114·13 | 119·20 | Spatial Lag Model | *ρ* = 0·233 | 115·13 | 121·88 | 1·003 | 0·317 |
|  |  |  |  |  |  |  | Spatial Error Model | *λ =* 0·189 | 115·61 | 122·37 | 0·517 | 0·472 |
| Innovative spirit  [GII] | 0·048 | 0·383 | 0·322 | 0·046 | 111·40 | 116·39 | Spatial Lag Model | *ρ* = 0·204 | 112·68 | 119·34 | 0·712 | 0·399 |
|  |  |  |  |  |  |  | Spatial Error Model | *λ =* 0·146 | 113·12 | 119·78 | 0·274 | 0·600 |

Note: Analyses were performed In Rv4·4·1 with the ‘rnaturalearth’, ‘sf’, ‘spdep’, and ‘spatialreg’ packages; Maximum Likelihood (ML) estimators; AIC = Akaike Information Criterion, BIC = Bayesian Information Criterion.
^1^The reference model for the Likelihood Ratio Test was the linear regression model (without spatial dependency).

The results revealed that there were no significant spatial autocorrelations (via Moran’s I test). The information criteria (AIC, BIC) descriptively favoured the linear regression models for all three significant predictors; also the spatial regression models, that additionally included a term for geospatial dependency, did not outperform the linear regression models (non-significant Likelihood Ratio tests). Following the law of parsimony, the modelling of associations should, therefore, be performed with the linear regression models.

## Supplementary Material 7. The robustness of the significant results in relation to differing operationalisations and calculations.

To examine the robustness of the associations between the three significant country-level predictors and curricular PL alignment at the time point of the study, we tested an alternative operationalisation or time point for the predictors. Effect size differences were calculated using the Psychometrica calculator (Lenhard & Lenhard, 2014) – Procedure #2: Testing the Significance of Correlations.

## For the association between educational attainment and curricular PL alignment

| **Main analysis**  (PISA 2022) | | | **Robustness analysis**  (Education subscore of HDI 2021) | | **Statistical test for differences**  **in the associative magnitude** | | | **Conclusion** |
| --- | --- | --- | --- | --- | --- | --- | --- | --- |
| *β* | *p* | 95% CI | *β* | *p* | ∆*β* | *z* | *p* | The association with the alternative indicator (education subscore of the HDI 2021) yielded a similar effect size, which was located within the confidence interval and did not statistically differ from the effect size of  the original operationalisation |
| 0·426 | 0·006 | 0·132, 0·651 | 0·392 | 0·012 | 0·034 | 0·249 | 0·402 |  |

Note: CI = confidence interval. The PISA 2022 and the education subscore of the HDI 2021 were strongly associated, *r* = 0·567.

## For the association between human development and curricular PL alignment

| **Main analysis**  (HDI 2021) | | | **Robustness analysis**  (HDI 2022) | | **Statistical test for differences**  **in the associative magnitude** | | | **Conclusion** |
| --- | --- | --- | --- | --- | --- | --- | --- | --- |
| *β* | *p* | 95% CI | *β* | *p* | ∆*β* | *z* | *p* | The association with the 2022 HDI (data was published after the discussion within the project consortium) yielded a similar effect size, which was located within the confidence interval and did not statistically differ from the effect  size of the original operationalisation |
| 0·322 | 0·043 | 0·011, 0·576 | 0·318 | 0·048 | 0·004 | 0·166 | 0·434 |  |

Note: CI = confidence interval. The HDI 2021 and the HDI 2022 were strongly associated, *r* = 0·988.

## For the association between innovation spirit and curricular PL alignment

| **Main analysis**  (GII 2023) | | | **Robustness analysis**  (European Innovation Score 2023) | | **Statistical test for differences**  **in the associative magnitude** | | | **Conclusion** |
| --- | --- | --- | --- | --- | --- | --- | --- | --- |
| *β* | *p* | 95% CI | *β* | *p* | ∆*β* | *z* | *p* | The association with the alternative indicator (the European Innovation Score) yielded a similar effect size, which was located within the confidence interval and did not statistically differ from the effect size of the original  operationalisation |
| 0·322 | 0·046 | 0·007, 0·579 | 0·348 | 0·032 | -0·026 | -0·366 | 0·357 |  |

Note: CI = confidence interval. No information was available for Greenland and Kazakhstan (missing data). The GII 2023 and the European Innovation Scoreboard 2023 were strongly associated, *r* = 0·900.

## Reference:

Lenhard, W. & Lenhard, A. (2014). Hypothesis Tests for Comparing Correlations. Available: https://[www.psychometrica.de/correlation.html.](http://www.psychometrica.de/correlation.html) Psychometrica. DOI: 10.13140/RG.2.1.2954.1367

## Supplementary Material 8. Comparison of linear with quadratic or cubic associations.

| **Predictors** | **Model** | **Model statistics** | | | | **Comparison with linear model^1^** | | **Final model selected** |
| --- | --- | --- | --- | --- | --- | --- | --- | --- |
|  |  | *F* | *df* | *p* | *R2* | *F* | *p* |  |
| Educational attainment [PISA] | Linear | 8·41 | 1, 38 | 0·006** | 0·181 | - | - | Linear |
|  | Quadratic | 4·10 | 2, 37 | 0·025* | 0·181 | 0·007 | 0·934 |  |
|  | Cubic | 2·76 | 3, 36 | 0·056 | 0·187 | 0·128 | 0·880 |  |
| Human development [HDI] | Linear | 4·39 | 1, 38 | 0·043* | 0·104 | - | - | Linear |
|  | Quadratic | 2·18 | 2, 37 | 0·127 | 0·106 | 0·081 | 0·777 |  |
|  | Cubic | 1·42 | 3, 36 | 0·254 | 0·106 | 0·042 | 0·961 |  |
| Economic strength [GDP per capita] | Linear | 0·987 | 1, 38 | 0·327 | 0·025 | - | - | Linear |
|  | Quadratic | 0·729 | 2, 37 | 0·489 | 0·038 | 0·485 | 0·491 |  |
|  | Cubic | 0·481 | 3, 36 | 0·698 | 0·039 | 0·247 | 0·782 |  |
| Societal liberty [HFI] | Linear | 2·41 | 1, 38 | 0·129 | 0·060 | - | - | Linear |
|  | Quadratic | 1·42 | 2, 37 | 0·256 | 0·071 | 0·455 | 0·504 |  |
|  | Cubic | 0·922 | 3, 36 | 0·440 | 0·071 | 0·277 | 0·798 |  |
| Innovative spirit [GII] | Linear | 4·28 | 1, 37 | 0·046* | 0·104 | - | - | Linear |
|  | Quadratic | 2·08 | 2, 36 | 0·139 | 0·104 | 0·003 | 0·959 |  |
|  | Cubic | 1·64 | 3, 35 | 0·123 | 0·123 | 0·390 | 0·680 |  |

Note: Following the law of parsimony, a more complex model should only be chosen in case of its statistical superiority; **p* < 0·05, ***p* < 0·01

^1^Nested model comparison (i.e., models differ only in the polynomic degree).

## Supplementary Material 9. The number of experts and population size per country.

| **Country** | **Number of representatives per country** | **Population^1^** |
| --- | --- | --- |
| Austria | 2 | 9,158,750 |
| Belgium | 2 | 11,817,096 |
| Bulgaria | 1 | 6,445,481 |
| Croatia | 2 | 3,850,894 |
| Cyprus | 2 | 921,000 |
| Czech Republic | 2 | 10,900,555 |
| Denmark | 2 | 5,961,249 |
| England | 2 | 57,180,000 |
| Estonia | 2 | 1,374,687 |
| Finland | 2 | 5,603,851 |
| France | 2 | 68,467,362 |
| Germany | 2 | 84,792,050 |
| Greece | 2 | 10,400,720 |
| Greenland | 1 | 56,657 |
| Hungary | 2 | 9,584,627 |
| Iceland· | 1 | 383,726 |
| Ireland | 2 | 5,201,740 |
| Italy | 2 | 58,971,230 |
| Kazakhstan | 1 | 20,133,000 |
| Latvia | 2 | 1,871,882 |
| Lithuania | 2 | 2,885,891 |
| Luxembourg | 1 | 672,050 |
| Montenegro | 2 | 619,100 |
| Netherlands | 2 | 17,942,942 |
| North Macedonia | 1 | 2,086,177 |
| Northern Ireland | 2 | 1,920,000 |
| Norway | 2 | 5,550,217 |
| Poland | 2 | 36,620,970 |
| Portugal | 2 | 10,639,726 |
| Romania | 2 | 19,054,548 |
| Scotland | 2 | 5,445,000 |
| Serbia | 2 | 6,664,177 |
| Slovakia | 1 | 5,424,687 |
| Slovenia | 1 | 2,123,949 |
| Spain | 2 | 48,619,695 |
| Sweden | 2 | 10,551,707 |
| Switzerland | 2 | 8,962,258 |
| Türkiye | 2 | 85,372,377 |
| Ukraine | 2 | 37,860,220 |
| Wales | 2 | 3,140,000 |
|  | ∑72 | ∑685,232,248 |

^1^References (Standing January 2024): Eurostat, ONS, Statistics Greenland, Statistics Iceland, Bureau of National Statistics Kazakhstan, National Statistical Office Montenegro, State Statistical Office North Macedonia, Statistics Norway, Statistical Office of Serbia, Swiss Federal Statistical Office, Turkish Statistical Institute, World Bank/UN

The 32 countries with two experts (average population size: *N* = 20,247,079) were significantly larger than the 8 countries with only one representative (average population size: *N* = 4,665,716): *t*(37.7) = -2.84, *p* = .004, *d* = -0.69. The countries that were represented by only one expert also had significantly lower educational attainment, *t*(38) = -3.09, *p* = .007, *d* = -1.12, and lower innovative spirit, *t*(37) = -2.04, *p* = .049, *d* = -0.85, but not lower human development, *t*(8.48) = -0.775, *p* = .459, compared to countries with two experts.

## Supplementary Material 10. Associations of ‘educational attainment’, ‘human development’, and ‘innovative spirit’ with single physical literacy indicators (item level).

| **Item** | **Indicator** | **Association with educational attainment (PISA)** | | **Association with human development (HDI)** | | **Association with innovative spirit  (GII)** | |
| --- | --- | --- | --- | --- | --- | --- | --- |
|  |  | *β* | *p* | *β* | *p* | *β* | *p* |
| i | Student-centred acting | 0·236 | 0·143 | 0·442** | 0·004 | 0·332* | 0·039 |
| ii | Development of a meaningful relationship with physical activities | 0·335* | 0·035 | 0·374* | 0·018 | 0·367* | 0·022 |
| iii | Acknowledgment of ‘unique journeys’ | 0·302 | 0·058 | 0·400* | 0·011 | 0·276 | 0·089 |
| iv | Balance between physical, cognitive, and psychosocial aspects | 0·097 | 0·551 | 0·136 | 0·404 | 0·120 | 0·467 |
| v | Exploration of different activity contexts | 0·312* | 0·050 | 0·071 | 0·663 | 0·147 | 0·371 |
| vi | Student involvement and provision of movement choice | 0·451** | 0·003 | 0·418** | 0·007 | 0·441** | 0·005 |
| vii | Embodiment and integration of body and mind | -0·101 | 0·537 | -0·140 | 0·388 | -0·030 | 0·856 |
| viii | Own responsibility for engagement in physical activities | 0·232 | 0·150 | 0·072 | 0·661 | 0·055 | 0·740 |
| ix | Availability of assessment/charting methods for individual progress | -0·082 | 0·614 | -0·254 | 0·114 | -0·106 | 0·522 |
| x | Transcendence of the current horizon through ‘lifelong learning’ | 0·091 | 0·579 | -0·060 | 0·712 | -0·004 | 0·980 |
| xi | Prioritisation of variety and exploration | 0·285 | 0·074 | 0·226 | 0·162 | 0·196 | 0·231 |
| xii | Reward and participation for everyone | 0·329* | 0·038 | 0·104 | 0·524 | 0·114 | 0·491 |
| xiii | Inclusion and accessibility of PE for all | 0·365* | 0·021 | 0·343* | 0·030 | 0·312 | 0·053 |
| xiv | Student-identified purpose for activities | 0·459** | 0·003 | 0·333* | 0·036 | 0·296 | 0·067 |
| xv | Encouragement of creativity and problem-solving | 0·466** | 0·002 | 0·376* | 0·017 | 0·334* | 0·037 |

Note: Unadjusted relationships between the three country-level predictors (in columns) and the 15 curricular physical literacy indicators; *p* < 0·05*, *p* < 0·01** PISA = Programme for International Student Assessment, HDI = Human Development Index, GII = Global Innovation Index.

## Supplementary Material 11. Sensitivity analysis after removing one remarkable outlier in the association between economic strength and curricular physical literacy alignment.

**
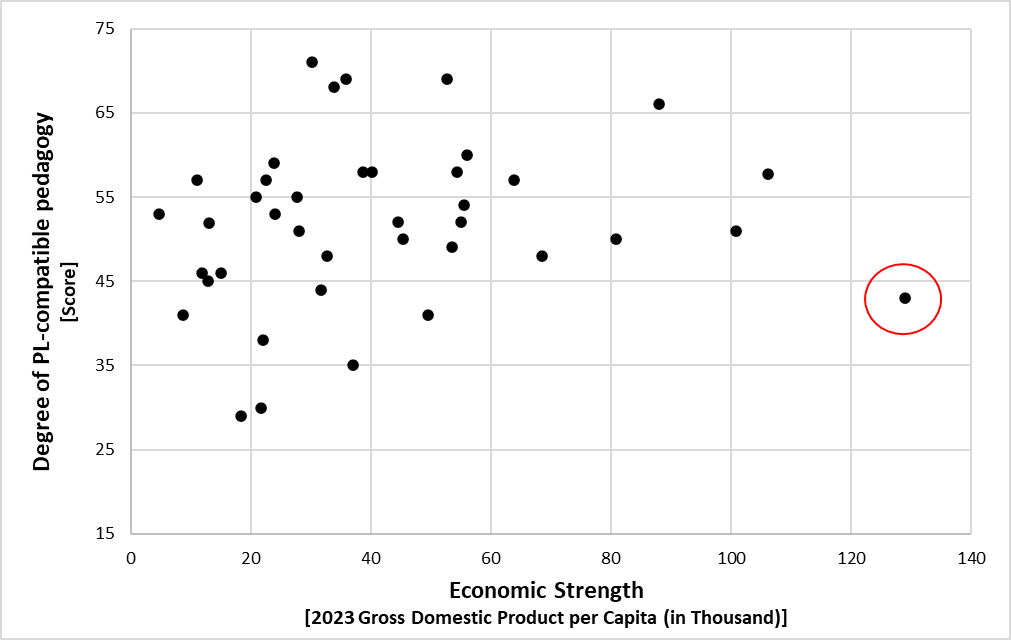
**

| **Main analysis**  (Without removal of outlier) | | | **Robustness analysis**  (After removal of outlier) | |
| --- | --- | --- | --- | --- |
| *β* | *p* | 95% CI | *β* | *p* |
| 0·159 | 0·327 | -0·160, 0·448 | 0·270 | 0·096 |

Luxembourg has an exceptionally high Gross Domestic Product (GDP) per capita of 128,936·00 US$ and may have substantially affected the association between economic strength and curricular PL alignment. Therefore, we have recalculated the association without this single data point. Despite increases in the associative magnitude from *β* = 0·159 to *β* = 0·270, the change was not significant (*z* = -0·498, *p* = 0·309) and, most importantly, the total association between economic strength and curricular PL alignment did not become significant.

## Supplementary Material 12. The regression residual *e* for each country differentiated by the three significant predictors showing significant associations with curricular physical literacy alignment.

| **Country** | **Educational attainment**  **[PISA]** | **Human development**  **[HDI]** | **Innovative spirit**  **[GII]** |
| --- | --- | --- | --- |
| Austria | 5·46 | 6·56 | 6·56 |
| Belgium | -2·54 | -2·68 | -0·49 |
| Bulgaria | 1·06 | -0·32 | -3·36 |
| Croatia | -22·94 | -20·03 | -18·82 |
| Cyprus | -8·47 | -17·27 | -16·46 |
| Czech Republic | -11·21 | -7·85 | -7·03 |
| Denmark | -7·21 | -7·33 | -7·02 |
| England | -14·34 | -13·21 | -15·08 |
| Estonia | 12·46 | 19·09 | 17·50 |
| Finland | 13·26 | 13·61 | 13·27 |
| France | -1·47 | -0·68 | -2·24 |
| Germany | -5·01 | -5·97 | -6·04 |
| Greece | 11·13 | 7·26 | 10·07 |
| Greenland | 2·79 | 2·67 | N/A |
| Hungary | -15·34 | -11·32 | -12·02 |
| Iceland· | 0·66 | -5·98 | -2·72 |
| Ireland | 0·79 | 2·58 | 5·10 |
| Italy | 4·66 | 5·79 | 6·46 |
| Kazakhstan | 7·40 | 4·68 | 6·10 |
| Latvia | 2·73 | 6·68 | 7·43 |
| Lithuania | -2·34 | -0·03 | 0·77 |
| Luxembourg | -5·27 | -11·27 | -9·69 |
| Montenegro | 2·26 | -2·50 | -0·15 |
| Netherlands | 3·26 | 2·08 | 1·50 |
| North Macedonia | 1·13 | -3·84 | -6·64 |
| Northern Ireland | 4·93 | 5·73 | 1·92 |
| Norway | 13·06 | 9·91 | 13·28 |
| Poland | -0·34 | 3·91 | 6·01 |
| Portugal | 1·53 | 4·50 | 3·94 |
| Romania | -17·81 | -18·85 | -19·13 |
| Scotland | -2·54 | -3·74 | -6·08 |
| Serbia | 8·33 | 10·27 | 9·33 |
| Slovakia | 2·19 | 3·56 | 4·44 |
| Slovenia | -6·41 | -5·56 | -2·28 |
| Spain | 14·66 | 15·20 | 16·66 |
| Sweden | -0·81 | -1·27 | -2·59 |
| Switzerland | -5·14 | -5·15 | -6·57 |
| Türkiye | -6·34 | -3·85 | -4·25 |
| Ukraine | 4·59 | 7·98 | 5·41 |
| Wales | 17·13 | 16·62 | 12·92 |

Note: N/A = No available data for this country (missing data).

The regression residual *e* results from subtracting the effective value of curricular PL alignment (*y*) from the expected value from the regression equation (^*y*). Positive regression residuals (*e*>0) denote higher PL alignment than expected by an indicator, while negative regression residuals (*e* < 0) describe lower PL alignment than expected by an indicator.
